# Does left ventricular function predict cardiac outcome in Anderson–Fabry disease?

**DOI:** 10.1007/s10554-020-02105-y

**Published:** 2020-11-19

**Authors:** Letizia Spinelli, Giuseppe Giugliano, Antonio Pisani, Massimo Imbriaco, Eleonora Riccio, Camilla Russo, Alberto Cuocolo, Bruno Trimarco, Giovanni Esposito

**Affiliations:** 1 Department of Advanced Biomedical Sciences, Federico II University of Naples, Via Pansini, 5, 80131 Naples, Italy; 2 Department of Public Health, Nephrology Unit, Federico II University, Naples, Italy

**Keywords:** Anderson–Fabry disease, Myocardial work, LV longitudinal strain, Adverse cardiac event

## Abstract

In Anderson–Fabry disease (AFD) the impact of left ventricular (LV) function on cardiac outcome is unknown. Noninvasive LV pressure–strain loop analysis is a new echocardiographic method to estimate myocardial work (MW). We aimed to evaluate whether LV function was associated with outcome and whether MW had a prognostic value in AFD. Ninety-six AFD patients (41.8 ± 14.7 years, 43.7% males) with normal LV ejection fraction were retrospectively evaluated. Inclusion criteria were sinus rhythm and ≥ 2-year follow-up. Standard echocardiography measurements, myocardial mechano-energetic efficiency (MEE) index, global longitudinal strain (GLS) and MW were evaluated. Adverse cardiac events were defined as composite of cardiac death, malignant ventricular tachycardia, atrial fibrillation and severe heart failure development. During a median follow-up of 63 months (interquartile range 37–85), 14 events occurred. Patient age, cardiac biomarkers, LV mass index, left atrium volume, E/Ea ratio, LV ejection fraction, MEE index, GLS and all MW indices were significantly related to adverse outcome at univariate analysis. After adjustment for clinical and echocardiographic parameters, which were significant at univariate analysis, GLS and MW resulted independent predictors of adverse events (p < 0.01). By ROC curve analysis, constructive MW ≤ 1513 mmHg% showed the highest sensitivity and specificity in predicting adverse outcome (92.9% and 86.6%, respectively). MW did not improve the predictive value of a model including clinical data, LV diastolic function and GLS. LV function impairment (both systolic and diastolic) is associated with adverse events in AFD. MW does not provide additive information over clinical features and systolic and diastolic function.

## Introduction

Anderson–Fabry disease (AFD) is an X-chromosome linked disorder caused by mutations in gene (GLA) coding for alpha-galactosidase-A enzyme [1]. Cardiac involvement is characterized by progressive accumulation of complex sphingolipids, predominantly globotriaosylceramide, resulting in myocardium thickening and fibrosis and abnormal left ventricular (LV) diastolic function [2], which represents the pathophysiologic substrate of heart failure symptoms. AFD related cardiomyopathy is the leading cause of mortality, accounting for 75% of all deaths [3, 4]. Although LV ejection fraction remains within normal until the late stages of the disease, impairment of LV function, as assessed by myocardial performance index, occurs in AFD patients [5]. Strain imaging has largely demonstrated reduction in LV global longitudinal strain (GLS) values even at early phase of cardiomyopathy [6–8]. There are limited data on the prognostic value of LV function in AFD patients [9]. In heart failure patients the assessment of GLS is superior to ejection fraction in predicting a poor outcome [10–13]. In patients with hypertrophic cardiomyopathy GLS and left atrial volume are independently associated with adverse outcome and may help to optimize risk stratification [14].

The assumption that LV systolic pressure corresponds to brachial cuff systolic blood pressure (SBP) allows to noninvasively calculate indices of LV function such as myocardial work (MW) and MW efficiency. A method based on LV longitudinal strain by speckle-tracking echocardiography and noninvasive standardized LV pressure curve has been introduced as a promising tool for noninvasive measurement of MW [15–18]. Likewise, LV myocardial mechano-energetic efficiency (MEE), which indicates the ratio between external work delivered by myocardium and the amount of total energy produced at each beat, can be estimated by means of a simple ultrasound-guided method [19, 20]. Low levels of MEE predict increased incidence of composite cardiovascular events in hypertensive patients [21].

In the present study we aimed to investigate the association between cardiac involvement evaluated with different echocardiographic measurements of LV function and adverse cardiac events in AFD patients with normal ejection fraction. In particular, we sought to evaluate whether there was any predictive value of standard echocardiography parameters, including LV diastolic function indices, of GLS and of complex measurements such as MEE and longitudinal strain-based MW.

## Methods

This retrospective analysis included all consecutive patients with genetically proved AFD and normal LV ejection fraction referred to our institution between October 2006 and October 2016, provided that they had three available echocardiographic apical views allowing LV longitudinal strain analysis and at least 2-year clinical follow-up. Exclusion criteria were as follows: history of coronary artery disease, moderate or severe valve heart disease, permanent atrial fibrillation, diabetes mellitus, signs of overt heart failure, complete left bundle branch block, past pacemaker/defibrillator and suboptimal echocardiographic image quality.

Ninety-six patients were included in the study (mean age 41.8 ± 14.7 years, 43.7% male gender). Data on clinical findings and adverse cardiac events were abstracted from the electronic patient record. In details, clinical data including New York Heart Association (NYHA) class, estimated glomerular filtration rate (eGFR), biomarkers values such as N-terminal brain natriuretic peptide (NT-proBNP) and high-sensitivity cardiac troponin I (hs-TnI) obtained within 2 weeks from echocardiography were utilized for the analysis. Chronic Kidney Disease Epidemiology Collaboration equation was used to calculate eGFR from serum creatinine values [22]. The Institutional Ethical Committee approved the study and written informed consent was obtained from all patients before the performed procedures. The study conformed to the principles of the Declaration of Helsinki on human research.

### Echocardiography analysis

The whole study population had two-dimensional transthoracic echocardiography, including color Doppler, pulsed-wave and continuous-wave Doppler and mitral annulus tissue Doppler at the initial evaluation. Echocardiography was performed with the use of a Vivid 7 ultrasound system (GE Healthcare, Horten, Norway) equipped with M4S transducer. Apical long-axis and two- and four-chamber views obtained with the highest possible frame rates (mean frame rate, 68 ± 9 frame per second (s)) were utilized to evaluate LV longitudinal strain. Cuff BP values, measured immediately at the end of echocardiogram with the patient in the recumbent position, were also obtained.

Standard echocardiography analysis was performed according to American Society of Echocardiography [23], as previously described [8]. Left atrial volume (area-length method in apical four-chamber and two-chamber views) was indexed to body surface area (left atrial volume index). LV mass was calculated by Devereux formula and normalized by height in meters to the power of 2.7. LV hypertrophy was defined as a value of LV mass index at least 47 g/m^2.7^ in women and at least 50 g/m^2.7^ in men, respectively [24].

LV end-diastolic (EDV) and end-systolic (ESV) volumes (from apical four-chamber and two-chamber views) were measured by the modified Simpson rule. Stroke volume (SV) was calculated as the difference between LVEDV and LVESV and ejection fraction was obtained as the ratio between SV and LVEDV. Stroke work (SW) was computed as echocardiographic SV times SBP, where SBP is the value of systolic BP taken by cuff sphygmomanometer immediately after echo exam [15]. MEE, which represents the ratio between SW and total energy consumption, was also calculated. Total energy consumption would ideally be determined by measurement of real-time myocardial oxygen consumption (MVO2) throughout coronary sinus catheterization or noninvasively with the use of PET techniques [14]. By assuming that MVO2 can be expressed by double product, i.e. heart rate (HR) and SBP product, we measured MEE according to following formulas:

$${\text{MEE (mL/s)}}\; = {\text{SW/MVO2}}\;{\text{ = }}\;{\text{SBP}} \times \;{\text{SV/BP}} \times {\text{HR}}\,{\text{ = }}\;{\text{SV/HR}}$$

where HR was expressed in beats/s. The normalization of MEE for LV mass (MEE index) provides the estimate of the ideal amount of blood pumped by each gram of LV mass in 1 s [20].

### Speckle tracking and myocardial work analysis

Speckle-tracking echocardiography analysis and strain-derived MW measurement were performed offline by using dedicated software (Echo Pac version BT12.0.0, GE Vingmed Ultrasound). Tracking of myocardial motion was performed with the region of interest adjusted to exclude the pericardium by attentively aligning the epicardial border. The integrity of tracking was visually confirmed as well as ascertained from the credibility of the strain curves, in addition to the automated tracking detection in the software. By analyzing LV according to a 17-segment model, GLS was calculated by averaging segmental longitudinal strain measured in 2-, 3-, and 4-chamber apical views. MW and related indices were obtained through a combination of LV longitudinal strain and non-invasively estimated LV pressure curves. Peak systolic LV pressure is assumed to be equal to the peak SBP measured from brachial cuff based noninvasive method immediately at the end of the echocardiographic study. The software can construct a non-invasive LV pressure curve adjusted according to the duration of the isovolumetric and ejection phases defined by the timing of aortic and mitral valve events by echocardiography. Strain and pressure data are then synchronized using the R wave on ECG as a common time reference. Global work index (GWI) was obtained as total work within the area of the pressure–strain loop, calculated from mitral valve closure to mitral valve opening. Moreover, additional indices of MW were calculated as follows: global constructive work (GCW) as the work performed during shortening in systole adding negative work during lengthening in isovolumetric relaxation; global wasted work (GWW) as the work performed during lengthening in systole and shortening in isovolumic relaxation and was associated with energy loss; global work efficiency (GWE) as the ratio between constructive work divided by the sum of constructive and wasted work. A single principal operator performed MW analysis in the present study. To assess the intra-observer and inter-observer reproducibility, MW measurements of 16 randomly selected patients were re-evaluated after 1 month from the initial analysis by the same principal operator blinded to the previous results and by a second observer blinded to the results of the principal operator. Intra-observer and inter-observer agreements were assessed by intraclass correlation coefficient (ICC) with 95% confidence interval (CI). The ICC chosen was of single measures and absolute agreement with random effect.

### Outcomes

Composite endpoint was the occurrence of fatal and non-fatal major adverse cardiac events as follows: (1) sustained ventricular tachycardia defined as consecutive beats arising below the atrioventricular node with an RR interval of < 600 ms and lasting ≥ 30 s, requiring hospital admission or identified by Holter monitoring, which was performed every one year, by internal protocol, or (2) newly diagnosed atrial fibrillation, or (3) severe heart failure defined as the development of New York Heart Association functional class III/IV symptoms, or (4) cardiac death, classified as sudden or heart failure related death. Patients without events were censored at the time of their last clinical follow-up. Medical records and death certificates of all patients who had an event were obtained and validated by a physician unaware of echocardiography evaluations. According to selection criteria, the minimum follow-up period was 24 months. For patients who had more than 1 event, only the first was considered for the analysis.

### Statistical analysis

Statistical analyses were performed using SPSS version 25.0 (Statistical Package for the Social Sciences, IBM, Chicago, Illinois, USA) and MedCalc version 12.7 (MedCalc Software Ltd, Ostend, Belgium). Variables were expressed as absolute number and percentage, mean ± SD, or median (interquartile range). Comparisons were made by χ^2^ or t test for unpaired samples, as appropriate.

Univariate and multivariate (entry criteria p < 0.05 at univariate analysis) Cox proportional hazard analyses were performed to verify if clinical, laboratory and imaging features were associated with the occurrence of cardiovascular events.

To identify the threshold level that provided the best cutoff for outcome prediction, we chose the value in which the sum of specificity and sensitivity was the highest. This value was obtained by receiver-operating characteristic (ROC) curve analysis. The C statistic was used to assess the ability to classify risk. Kaplan–Meier survival curves and the log-rank test were used for visualization of cumulative event-free survival and estimate of probability values.

The incremental value of LV function as assessed by MEE index, GLS and MW indices in addition to clinical factors and standard echocardiography parameters was assessed with the use of the Cox proportional hazard model and a stepwise fashion.

All statistical tests were 2-sided. For all tests, a p value < 0.05 was considered statistically significant.

## Results

Clinical and imaging characteristics of the entire study population and according to sex are summarized in Table 1. Male and female patients were of the same age, however, as expected, male patients had worse renal function and were more prone to have LV hypertrophy. Four male patients had history of renal transplantation and one of them had undergone kidney transplant before being diagnosed with AFD.

**Table 1:** Characteristics of AFD patients according to sex

	All patients (n = 96)	Females (n = 54)	Males (n = 42)	p
Age (years)	41.8 ± 14.7	41.1 ± 14.9	42.8 ± 14.6	0.570
BMI (Kg/m^2^)	25.5 ± 5.2	25.2 ± 6.0	25.7 ± 3.8	0.578
NYHA class 1	84 (87.5)	49 (90.7)	35 (83.3)	0.276
NYHA class 2	12 (12.5)	5 (9.3)	7 (16.7)	0.276
eGFR (mL/min/1.73 m^2^ )	93.2 ± 31.9	102.2 ± 25.4	81.6 ± 35.7	0.001
Kidney transplant, n (%)	4 (4.2)	0	4 (9.5)	< 0.001
ERT at baseline, n (%)	16 (16.7)	4 (7.4)	12 (28.6)	0.006
ERT started during follow-up, n (%)	59 (61.5)	23 (42.6)	36 (85.7)	< 0.001
Heart rate (b/min)	69.0 ± 11.5	70.2 ± 11.1	67.4 ± 11.9	0.235
SBP (mmHg)	123.0 ± 16.6	119.8 ± 16.1	127.0 ± 16.6	0.034
DBP (mmHg)	74.9 ± 10.7	74.2 ± 11.4	75.8 ± 9.9	0.452
Hs-TnI (pg/mL)	33.9 ± 111.9	21.7 ± 65.1	49.5 ± 151.9	0.228
NT-proBNP (pg/mL)	352.4 ± 769.6	184.7 ± 496.4	568.1 ± 984.4	0.015
LV hypertrophy, n (%)	35 (36.5)	11 (20.4)	24 (57.1)	< 0.001
LV mass index (g/m^2.7^)	48.5 ± 19.7	42.3 ± 15.6	57.8 ± 27.9	< 0.001
Left atrium volume (mL/m^2^)	36.5 ± 14.7	33.9 ± 15.4	39.8 ± 13.2	0.050
E/Ea ratio	9.8 ± 4.8	8.9 ± 4.1	10.9 ± 5.4	0.037
LV EDVi (mL/m^2^)	59.1 ± 15.6	55.6 ± 15.9	63.5 ± 14.3	0.014
LV ESVi (mL/m^2^)	23.5 ± 11.2	21.8 ± 10.9	25.6 ± 11.3	0.099
LV ejection fraction (%)	62.4 ± 5.8	63.2 ± 5.8	61.4 ± 5.8	0.126
GLS (%)	-16.9 ± 4.1	-18.2 ± 3.8	-15.3 ± 3.9	< 0.001
MEE index (mL/s per g)	0.32 ± 0.12	0.33 ± 0.12	0.31 ± 0.13	0.394
GWI (mmHg%)	1678.4 ± 523.61	1815.4 ± 506.2	1502.2 ± 497.5	0.003
GCW (mmHg%)	1821.6 ± 546.0	1953.9 ± 540.1	1651.4 ± 510.9	0.006
GWW (mmHg%)	99.3 ± 77.2	94.7 ± 82.4	105.1 ± 70.5	0.517
GWE (%)	92.5 ± 6.8	93.8 ± 6.1	91.0 ± 7.5	0.047

*BMI* body mass index, *eGFR* estimated glomerular filtration rate, *ERT* enzyme replacement therapy, *SBP* systolic blood pressure, *DBP* diastolic blood pressure, *hs-TnI* high-sensitivity troponin I, *NT-proBNP* N-terminal prohormone of Brain Natriuretic Peptide, *LV* left ventricular, *E/Ea ratio* the ratio of mitral (E) to mitral annulus (Ea) early diastolic peak velocity, *EDVi* end-diastolic volume indexed to body surface area, *ESVi* end-systolic volume indexed to body surface area, *MEE* mechano-energetic efficiency, *GWI* global work index, *GCW* global constructive work, *GWW* global wasted work, *GWE* global work efficiency

Sixteen patients (16.6%) were treated with enzyme replacement therapy (ERT) at baseline evaluation, with mean treatment duration of 6.0 ± 4.1 years, while 59 patients (61.4%) initiated ERT during follow-up. Patently, the percentage of patients receiving ERT was higher among male than female patients, either at first evaluation or during follow-up. Eighty-four patients were classified as NYHA class 1 and 12 patients as NYHA class 2.

**Fig. 1:**
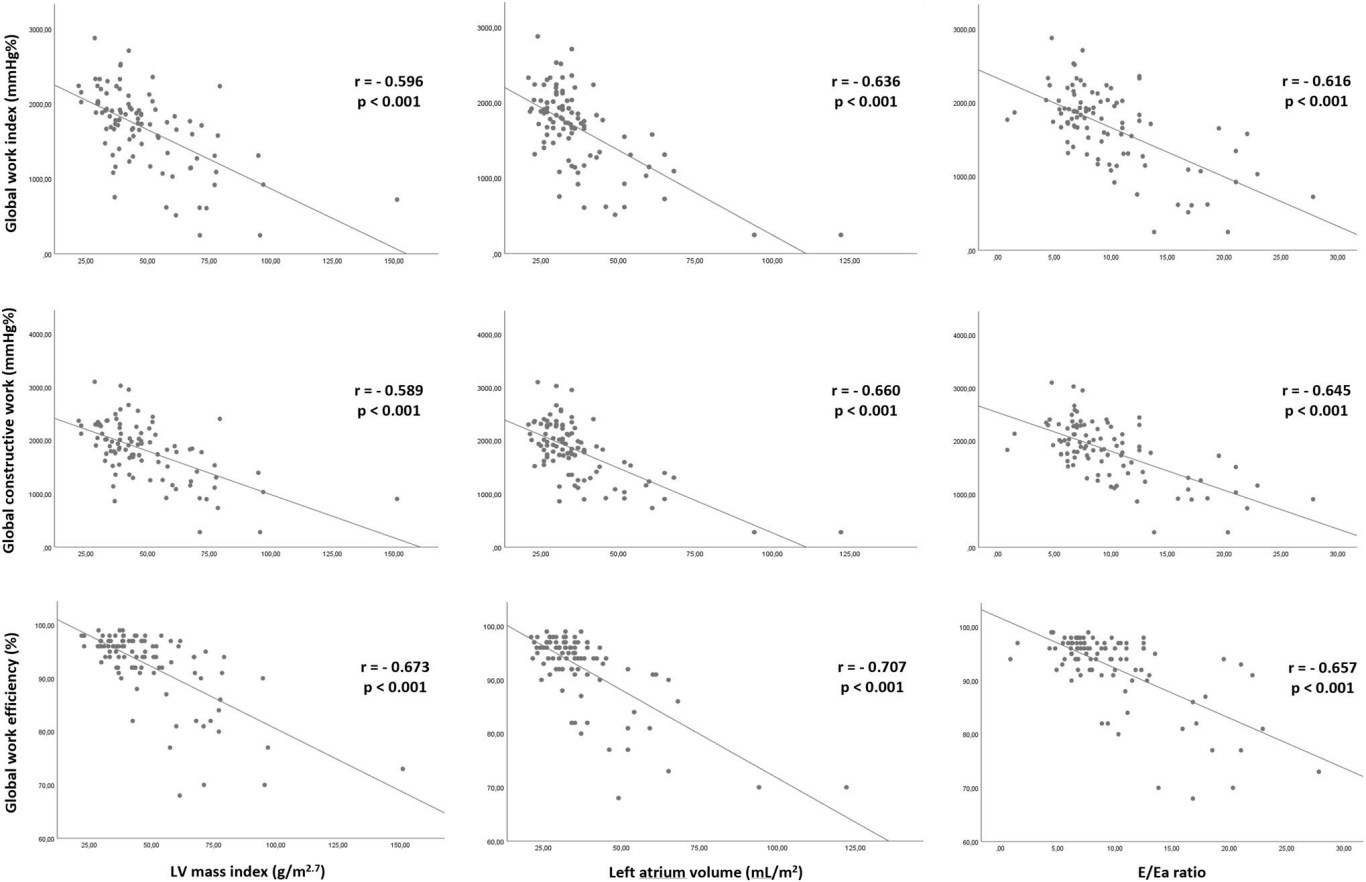
Relationships between LV myocardial work indices and standard echocardiography parameters. Linear regression analysis showing the relationship between LV mass index, left atrial volume and E/Ea ratio with global work index (upper panel), global constructive work (middle panel), and global work efficiency (lower panel)

Male patients had worse LV GLS as compared to females, but similar MEE index values. Although no difference in GWW between male and female patients was observed, GCW, GWI and GWE were significantly lower in males (Table 1). The latter myocardial work indices showed a statistically significant correlation with LV mass, left atrium volume and E/Ea ratio (Fig. 1). GLS and MEE index were also significantly correlated with LV mass (r = − 0.561, p < 0.001 and r = − 0.588, p < 0.001, respectively), left atrium volume (r = − 0.465, p < 0.001, and r = − 0.508, p < 0.001, respectively) and E/Ea ratio (r = − 0.415, p < 0.001, and r = − 0.516, p < 0.001, respectively).

Of note, the intra-observer agreement between 2 repeated evaluations of MW work was excellent for all the parameters (for GWE, ICC = 0.990, 95% CI 0.972–0.997; for GWI, ICC = 0.947, 95% CI 0.858–0.981; for GCW, ICC = 0.947, 95% CI 0.858–0.981 and for GWW, ICC = 0.952, 95% CI 0.870–0.983). The inter-observer agreement was also good to excellent (for GWE, ICC = 0.967, 95% CI 0.909–0.988; for GWI, ICC = 0.871, 95% CI 0.671–0.953; for GCW, ICC = 0.835, 95% CI 0.588–0.939 and for GWW, ICC = 0.831, 95% CI 0.580–0.938).

During a median follow-up of 63 months (interquartile range 37–85 months), 14 adverse cardiac events were detected. Two patients experienced severe heart failure and 2 patients heart failure-related death (a 73 years-old woman who had been under ERT during the last 10 years and a 71 years-old woman who never received ERT, respectively). Six patients had implanted cardioverter-defibrillator for sustained ventricular tachyarrhythmia. Of note, two of them died within the first year after implant. They were a 55 years-old man who had been under ERT over the last 12 years and a 50 years-old man who had been diagnosed with AFD at age of 40 years, respectively. Four patients developed permanent atrial fibrillation. Seven out of 14 patients experiencing adverse cardiac events had been classified as NYHA class 2.

Table 2 displays clinical and echocardiographic predictors of adverse cardiac events during follow-up at univariate Cox regression analysis. The following factors were significantly related to a poor outcome: patient age, eGFR, cardiac biomarkers, LV hypertrophy, LV mass index, left atrium volume, E/Ea ratio, LV ejection fraction, GLS, MEE index, and all MW indices. Multivariate Cox regression analyses were performed by including all clinical and standard echocardiography variables found significantly associated with a poor outcome at univariate analysis and, time by time, GLS, MEE index, and each MW parameter. After adjustment for clinical and standard echocardiography variables, GLS and all MW indices resulted independently associated with adverse cardiac events, while MEE index values did not (Table 3).

**Table 2:** Predictors of events during follow-up at univariate Cox analysis

	HR	95% CI	p
Age (years)	1.082	1.034–1.133	0.001
Male sex	2.346	0.783–7.033	0.128
BMI (Kg/m^2^)	1.019	0.938–1.107	0.656
eGFR (mL/min/1.73 m^2^)	0.978	0.961–0.996	0.016
Heart rate (b/min)	0.989	0.943–1.037	0.647
SBP (mmHg)	1.008	0.974–1.043	0.668
DBP (mmHg)	1.010	0.967–1.054	0.657
hs-TnI (pg/mL)	1.002	1.001–1.004	0.031
NT-proBNP (pg/mL)	1.001	1.001–1.002	0.002
LV hypertrophy	13.805	1.802–105.750	0.012
LV mass index (g/m^2.7^)	1.022	1.008–1.036	0.002
Left atrium volume (mL/m^2^)	1.028	1.011–1.045	0.001
E/Ea ratio	1.162	1.082–1.248	< 0.001
LV EDVi (mL/m^2^)	1.006	0.974–1.038	0.730
LV ESVi (mL/m^2^)	1.021	0.993–1.048	0.139
LV ejection fraction (%)	0.926	0.873–0.984	0.012
Global longitudinal strain (%)	1.309	1.124–1.525	0.001
MEE index (mL/s per g)	0.906	0.849–0.965	0.002
GWI (mmHg%)	0.998	0.997–0.999	< 0.001
GCW (mmHg%)	0.998	0.997–0.999	< 0.001
GWW (mmHg%)	1.006	1.003–1.009	< 0.001
GWE (%)	0.897	0.854–0.943	< 0.001

*HR* hazard ratio, *CI* confidence interval, *eGFR* estimated glomerular filtration rate, *hs-TnI* high-sensitivity troponin I, *NT-proBNP* N-terminal prohormone of Brain Natriuretic Peptide, *LV* left ventricular, *E/Ea ratio* the ratio of mitral (E) to mitral annulus (Ea) early diastolic peak velocity, *EDVi* end-diastolic volume indexed to body surface area, *ESVi* end-systolic volume indexed to body surface area, *MEE* mechano-energetic efficiency, *GWI* global work index, *GCW* global constructive work, *GWW* global wasted work, *GWE* global work efficiency

**Table 3:** Univariate and multivariate Cox regression analysis of the ability of complex LV function measurements to predict adverse cardiac events

	Global longitudinal strain (%)			MEE index (mL/s per g)			GWI (mmHg%)		
	HR	95% CI	p	HR	95% CI	p	HR	95% CI	p
Univariate analysis	1.309	1.124–1.525	0.001	0.906	0.849–0.965	0.002	0.998	0.997–0.999	< 0.001
Adjusted model^a^	1.373	1.035–1.821	0.028	0.942	0.850–1.043	0.249	0.997	0.996–0.999	0.003

	GCW (mmHg%)			GWW (mmHg%)			GWE (%)		
	HR	95% CI	p	HR	95% CI	p	HR	95% CI	p
Univariate analysis	0.998	0.997–0.999	< 0.001	1.006	1.003–1.009	< 0.001	0.897	0.854–0.943	< 0.001
Adjusted model^a^	0.997	0.995–0.999	0.010	1.012	1.004–1.020	0.003	0.820	0.725–0.927	0.002

*HR* hazard ratio, *CI* confidence interval, *MEE* mechano-energetic efficiency, *GWI* global work index, *GCW* global constructive work, *GWW* global wasted work, *GWE* global work efficiency

^a^Multivariate Cox analyses adjusted for age, estimated glomerular filtration rate, high-sensitivity troponin I, N-terminal prohormone of Brain Natriuretic Peptide, left ventricular mass index, left atrium volume, the ratio of mitral to mitral annulus early diastolic peak velocity, and left ventricular ejection fraction (entry criteria p < 0.05 at univariate analysis)

**Fig. 2:**
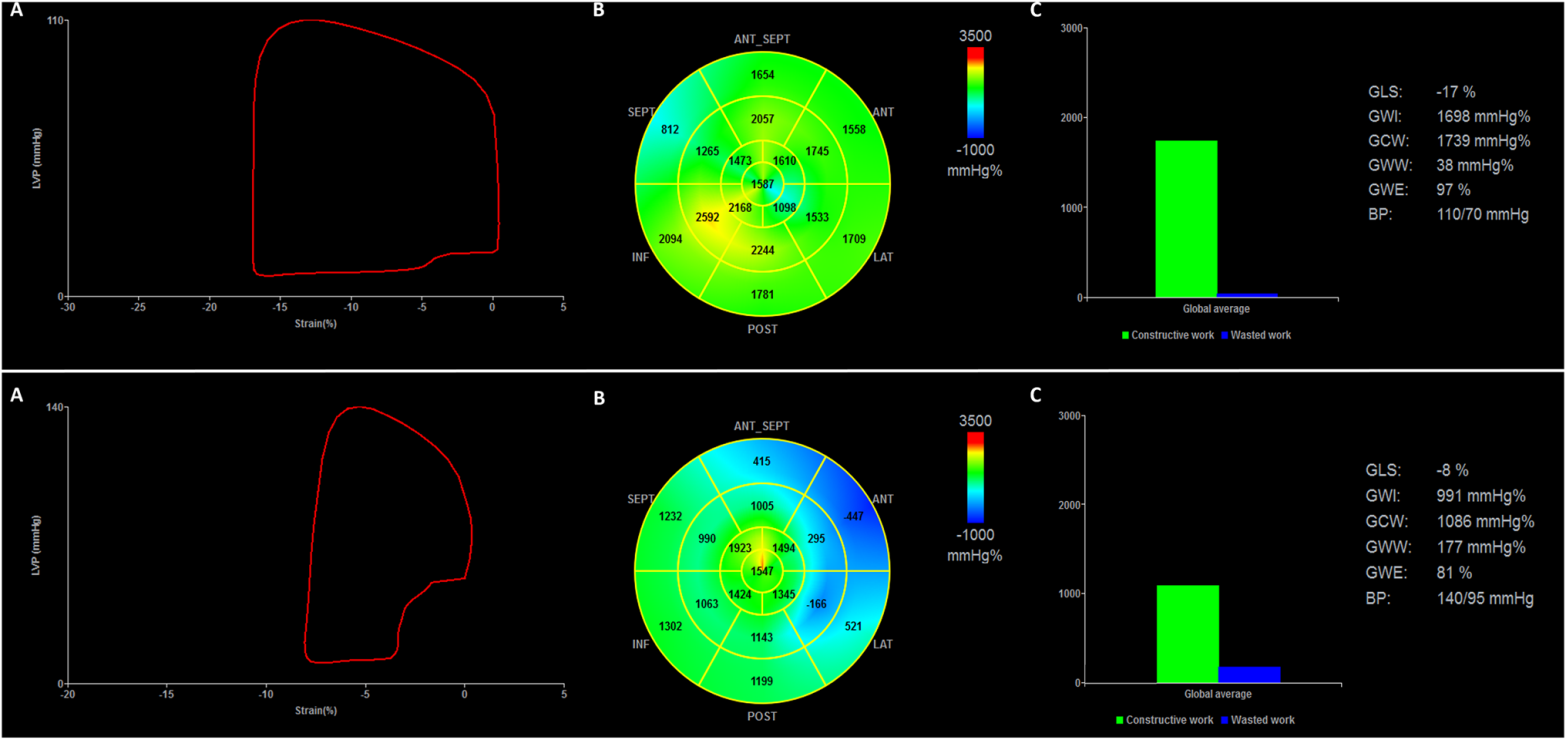
Pressure–strain loops by estimated LV pressure and LV longitudinal strain by means of echocardiography. Representative example of global myocardial work measurements from an event-free AFD patient (upper panel) and a patient with adverse event at follow-up (lower panel). **a** LV pressure–strain loop; **b** Bull’s eye plot showing segmental LV myocardial work index in a 17-segment model; **c** constructive work (green column) and wasted work (blue column) values. *LVP* LV pressure, *GLS* global longitudinal strain, *GWI* global work index, *GCW* global constructive work, *GWW* global wasted work, *GWE* global work efficiency, *BP* blood pressure

Figure 2 displays a representative example of MW measurements from an event-free AFD patient and a patient with adverse event at follow-up, respectively.

**Fig. 3:**
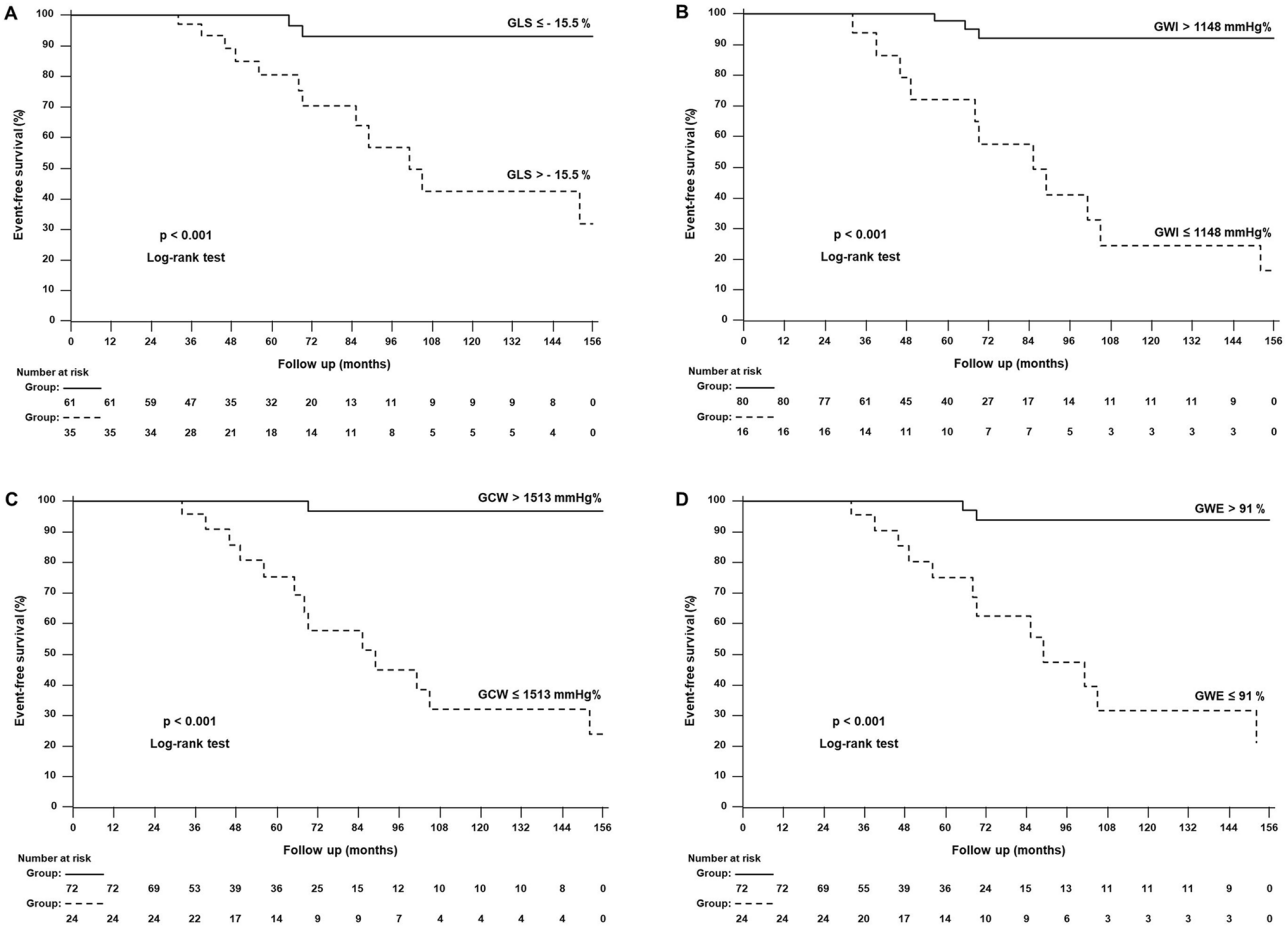
Global longitudinal strain, myocardial work indices and prognosis. Kaplan–Meier curves for major cardiac events in AFD patients categorized according to the cut-off values obtained by ROC analysis for **a** global longitudinal strain (GLS), **b** global work index (GWI), **c** global constructive work (GCW) and **d** global work efficiency (GWE)

Table 4 shows the accuracy in predicting outcome of clinical, laboratory and echocardiographic features by ROC curve analysis. Interestingly, all MW indices exhibited high accuracy, with GCW having the highest area under the curve and Youden index; the GCW cut-off of ≤ 1513 mmHg% showed a sensitivity of 92.9% and a specificity of 86.6% in predicting adverse cardiac outcome. Conversely, sensitivity and specificity for GLS > − 15.5% were 85.7% and 71.9%, respectively. Figure 3 displays the Kaplan–Meier survival probability curves of AFD patients categorized according to the cut-offs obtained by ROC analysis for GLS (> − 15.5%), GWI (≤ 1148 mmHg%), GCW (≤ 1513 mmHg%) and GWE (≤ 91%).

**Table 4:** Predictors of events accuracy (ROC curve analysis)

	AUC	95% CI	p	Youden index	Criterion	Sensitivity %	Specificity %
Age (years)	0.767	0.669–0.847	0.0001	0.4460	> 49	71.4	73.2
Male sex	0.620	0.515–0.717	0.0942	0.2404	+	64.3	59.8
eGFR (mL/min/1.73 m^2^)	0.749	0.650–0.832	0.0003	0.4007	≤ 69.0	57.1	82.9
hs-TnI (pg/mL)	0.878	0.795– 0.936	< 0.0001	0.7596	> 8.0	85.7	90.2
NT-proBNP (pg/mL)	0.834	0.744–0.902	< 0.0001	0.5906	> 158	78.6	80.5
LV hypertrophy	0.830	0.745–0.915	< 0.0001	0.6603	+	92.9	73.2
LV mass index (g/m^2.7^)	0.872	0.744–1.000	< 0.0001	0.7700	> 54.3	92.9	84.1
Left atrium volume (mL/m^2^)	0.909	0.819–0.999	< 0.0001	0.7247	> 43	78.6	93.9
E/Ea	0.925	0.845–1.000	< 0.0001	0.8084	> 12.8	85.7	95.1
LV ejection fraction (%)	0.708	0.512–0.904	0.0372	0.4617	≤ 57	57.1	89.0
GLS (%)	0.848	0.756–0.940	< 0.0001	0.5767	> -15.5	85.7	71.9
MEE index (mL/s per g)	0.858	0.740–0.977	< 0.0001	0.6969	≤ 0.26	92.9	76.8
GWI (mmHg%)	0.892	0.766- 1.000	< 0.0001	0.7247	≤ 1148	78.6	93.9
GCW (mmHg%)	0.908	0.797–1.000	< 0.0001	0.7944	≤ 1513	92.9	86.6
GWW (mmHg%)	0.812	0.697–0.928	< 0.0001	0.4669	> 83	85.7	61.0
GWE (%)	0.915	0.830–1.000	< 0.0001	0.7108	≤ 91	85.7	85.4

*ROC* receiver operating characteristic, *AUC* area underthecurve, *CI* confidence interval, *eGFR* estimated glomerular filtration rate, *hs-TnI* high-sensitivity troponin I, *NT-proBNP* N-terminal prohormone of Brain Natriuretic Peptide, *LV* left ventricular, *E/Ea ratio* the ratio of mitral (E) to mitral annulus (Ea) early diastolic peak velocity, *MEE* mechano-energetic efficiency, *GWE* global work efficiency, *GWI* global work index, *GCW* global constructive work, *GWW* global wasted work

**Fig. 4:**
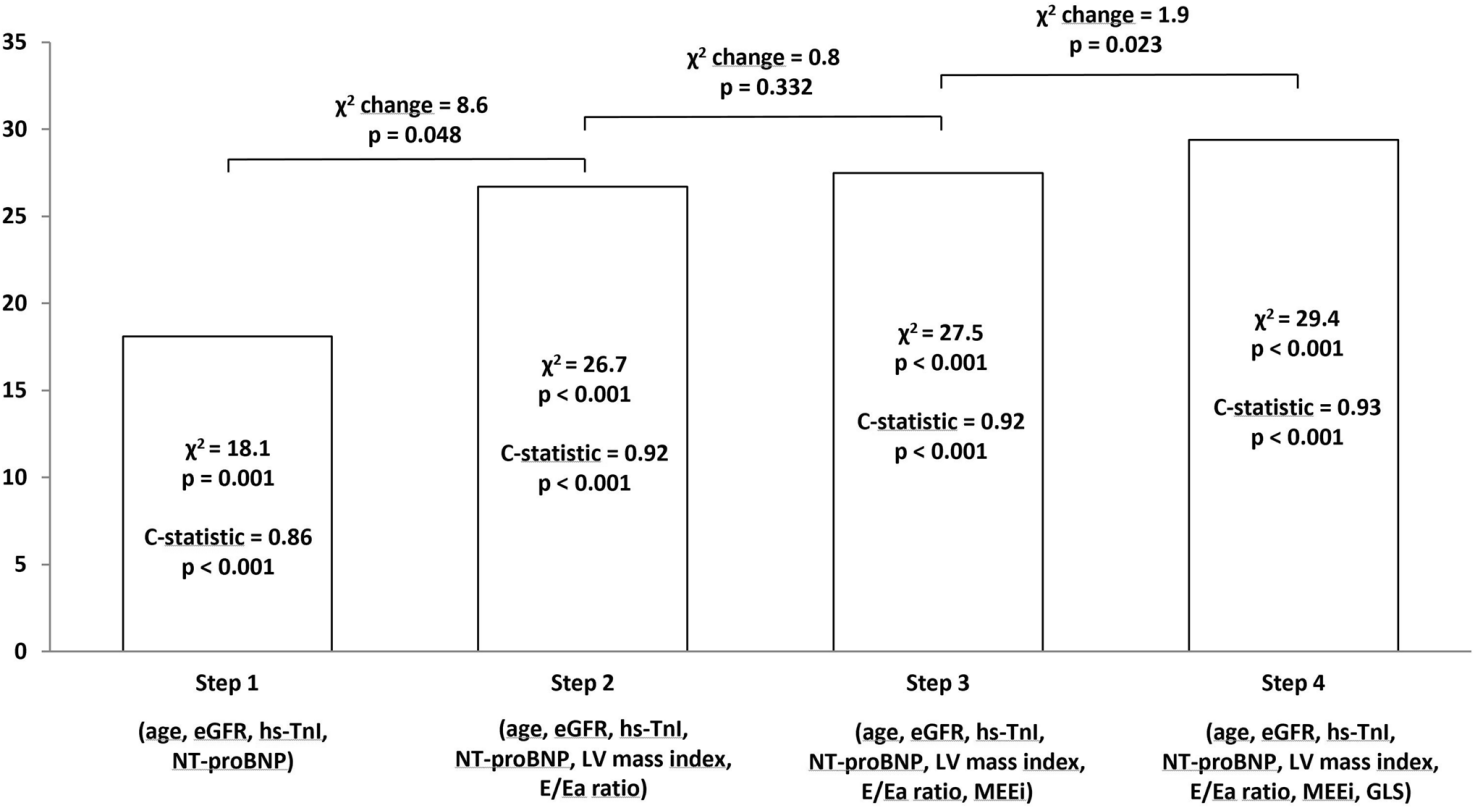
Incremental value in predicting outcome of global longitudinal strain over clinical and traditional echocardiographic variables. Bars show the global χ^2^ values of different prediction models obtained by Cox regression analysis in a stepwise fashion

By Cox regression analysis, we found that both LV mass and diastolic dysfunction showed an additive prognostic value in detecting AFD patients at risk over a model containing clinical features such as patient age, eGFR, NT-proBNP and hs-TnI levels (Fig. 4). The model did not further improve by adding MEE index. Conversely, the subsequent addition of GLS provided a modest but significant incremental value in predicting outcome (Fig. 4). Moreover, any MW parameters did not provide significant incremental value in outcome prediction when added to clinical information, standard echocardiography parameters and GLS.

## Discussion

The main results of the present study including AFD patients with normal LV ejection fraction can be summarized as follows: (1) both LV systolic function impairment and diastolic dysfunction are associated to the risk of adverse cardiac events; (2) after adjustment for clinical and standard echocardiography variables, LV GLS and MW indices are independently associated with adverse cardiac outcome; (3) any MW parameter does not improve the predictive value of a model including clinical features, LV mass assessment, LV diastolic function and GLS.

According to Fabry registry data, cardiac events are the most common adverse events in AFD patients [4, 25], even in those under ERT [26], followed by renal events, stroke, and non-cardiac death. Indeed, life expectancy is reduced in AFD, as death occurs at a mean age of 55 years in men and at 66 years in women [4]. Patients with adverse cardiac events show more advanced cardiac and renal disease as compared with patients without events.

Our study supports LV hypertrophy as a risk factor associated with cardiovascular events [25, 26] and, for the first time, attempts to shed some light on the predictive value of LV function in AFD patients with normal ejection fraction. Findings suggest that LV diastolic dysfunction and impairment of systolic function, as measured by GLS and strain derived MW, are relevant for prognosis assessment in this clinical setting. The independent prognostic value of strain-related parameters was maintained even after adjustment for clinical and standard echocardiography variables. GLS is a reliable marker of LV systolic function in many clinical conditions [10–13, 27].

The incremental value, albeit modest, of GLS over LV mass and diastolic function in addition to clinical risk factors, claims a role for LV systolic longitudinal function in determining cardiac prognosis of AFD patients. In principle, MW, as assessed by noninvasive LV pressure–longitudinal strain curves, is superior to myocardial strain in evaluating LV systolic function. It indeed takes in account deformation as well as afterload and strictly correlates with the equivalent invasive work measurement, as demonstrated in both experimental and clinical studies [15, 16]. In the present AFD patient cohort, we observed a higher accuracy of MW parameters in comparison with GLS in predicting event free survival, with GCW being the best performing index. Indeed, GCW measurement includes work performed during shortening in systole adding negative work during lengthening in isovolumetric relaxation, both active processes influencing oxygen consumption. Consequently, GCW is related to exercise capacity in heart failure patients with preserved ejection fraction [28], as well as in patients with hypertrophic cardiomyopathy [29]. Our findings are in line with studies demonstrating association between MW and adverse cardiac outcome in patients with cardiac amyloidosis or nonobstructive hypertrophic cardiomyopathy [30, 31]. In patients suffering from cardiac amyloidosis a superior prognostic value of MW to GLS has been demonstrated for prediction of adverse cardiac events and all-cause mortality [30]. Likewise, Hiemstra and colleagues found a significant association of GCW with adverse events in patients with nonobstructive hypertrophic cardiomyopathy [31]. However, for the first time we sought to explore the additive value of MW over LV diastolic function and we found that adding mechanical indices to a model including clinical variables, LV mass, E/Ea and GLS did not improve the predictive value. In our opinion, the remarkably high correlation found between E/Ea and MW indices in this cohort of AFD patients might at least partially account for results. Yet, in normal subjects as well as in patients with nonobstructive hypertrophic a poor correlation has been documented between LV diastolic function and MW indices [17, 31]. The association between LV diastolic dysfunction and MW impairment could be explained by pathogenetic mechanisms underlying AFD related cardiac involvement. Heart cells storage of glycosphingolipids, indeed, results in cellular structural changes and functional disruption, which act as stimuli for a cascade of events including oxidative stress, impairment in mitochondrial function, tissue inflammatory response, endothelial cells dysfunction [32–34]. Decreased activities of several mitochondrial respiratory chain complexes were demonstrated in fibroblasts from AFD patients, which were reflected in decreased cellular levels of ADP and creatinephosphate [32]. These findings are of relevance in AFD patients in vivo as reduced levels of creatinephosphate and ATP were found in heart. All the effects contribute to impair myocardial contractility and to delay ventricular relaxation. Disease-specific mechanisms for myocardial dysfunction are strongly suggested by the evidence of myocardial deformation impairment before the occurrence of LV hypertrophy [8, 35]. A reduction of mitral annulus systolic and relaxation tissue Doppler velocities regardless of LV mass has been demonstrated in AFD patients [36]. Along with subclinical systolic function impairment, reduced longitudinal strain rate during isovolumic diastolic period has been found in AFD patients before the development of LV hypertrophy [37]. We found lower MW indices with the increase of LV mass and the worsening of LV diastolic function. Pathogenetic mechanisms underlying LV dysfunction and cardiac hypertrophy development in AFD are likely to explain the association between LV mass increase and impairment of MW. However, similar results were previously descried in patients with hypertrophic cardiomyopathy [29] and in patients with dilated cardiomyopathy [38]. At variance, in uncontrolled hypertensive patients a positive correlation was documented between indices of MW and LV mass to match arterial afterload and allow preservation of ejection fraction [38].

It is conceivable that intrinsic impairment of myocardial function might impact on the prognosis of AFD patients. Our findings seem to indicate that LV longitudinal strain as well as strain-derived MW can foresee risky condition more accurately than stroke volume based functional measurements, given that MEE index, which was associated with adverse events at univariate analysis, lost any predictive value at multivariate analysis including clinical and standard echocardiography parameters. Longitudinal strain indeed reflects intrinsic myocardial function more closely than measurements based on traditional parameters as it evaluates the active component of deformation [39]. However, the limited value of stroke volume calculation based on 2D volumes should be acknowledged.

Even with caution due to the relatively small sample size, our results indicate that the use of a cluster of factors such as patient age, cardiac biomarkers, LV mass, diastolic dysfunction and GLS strain can provide valuable criteria for identification of risky AFD patients.

By exploring the additive prognostic value of MW over demographic and bio-humoral data and standard echocardiography measurements, we were able to demonstrate that MW indices can identify with high accuracy risky patients in the setting of AFD and provide information comparable to that obtained by clinical features, LV hypertrophy and diastolic function. This is an undoubtably important issue as MW assessment is not available from most vendors.

## Limitations

Several potential limitations should be acknowledged. Given the single center nature of the study and the rarity of disease, the number of included patients can be considered small. Although comparable to other studies addressing cardiac prognosis in AFD [40, 41], the small sample size limits the quality of the statistical tests and may have resulted in underpowered tests. Additionally, a composite endpoint was used to assess cardiac risk, as the number of expected events was modest. Given that the expected effects on each individual event were similar, on the basis on biologic plausibility, use of composite endpoint could be considered as justified.

The use of assumptions and composite measurements to obtain some complex echocardiographic parameters might represent another potential limit for interpretation of the results. Indeed, measurement errors may accumulate by successive approximations and assumptions. Also, MW should preferably be calculated by stress-strain loop area and not by pressure–strain loop area. Given that the risky group had increased LV wall thickness the actual MW might be lower than what is reported in our study. Furthermore, we acknowledge that results reflect a best-case scenario as we calculated optimal cut-off points and tested these in the same AFD patient cohort. It would be preferably to externally validate the calculated cut-off points in a larger independent cohort of AFD patients.

A further potential limitation might arise from the fact that the potential predictive information provided by myocardial fibrosis had not been addressed, since cardiac magnetic resonance was not available for every patient included in the study. However, studies utilizing analysis of myocardial late gadolinium enhancement (LGE) in AFD patients found that more extensive LGE certainly places patients in a higher-risk category, but that the presence of LGE alone does not necessarily imply a poor outcome [40, 41]. Moreover, a consistent percentage (21%) of patients who experienced an adverse event did not have LGE [41]. The sensitivity and specificity we found in our AFD cohort for GCW as outcome predictor were higher of those reported for LGE, claiming a role for this functional parameter in detecting patients with poor prognosis.

## Conclusions

Results of the present study demonstrate that echocardiography is a risk stratification tool to assess prognosis in AFD patients with normal LV ejection fraction. Firstly, LV diastolic dysfunction in association with demographic data and cardiac biomarkers can depict an adverse risk profile for cardiac events. Secondly, GLS and MW appear to be good predictors of poor cardiac outcome. However, data need to be further explored in larger AFD populations, potentially by multicenter studies. If confirmed, they would provide clinicians useful tools in AFD management to delineate risk profile and address a patient tailored treatment.
